# Measurement of Tongue Tip Velocity from Real-Time MRI and Phase-Contrast Cine-MRI in Consonant Production

**DOI:** 10.3390/jimaging6050031

**Published:** 2020-05-13

**Authors:** Karyna Isaieva, Yves Laprie, Freddy Odille, Ioannis K. Douros, Jacques Felblinger, Pierre-André Vuissoz

**Affiliations:** 1 Université de Lorraine, INSERM, IADI, F-54000 Nancy, France; freddy.odille@inserm.fr (F.O.); ioannis.douros@loria.fr (I.K.D.); j.felblinger@chru-nancy.fr (J.F.); pa.vuissoz@chru-nancy.fr (P.-A.V.); 2 Université de Lorraine, CNRS, Inria, LORIA, F-54000 Nancy, France; Yves.Laprie@loria.fr; 3 CIC-IT, INSERM, CHRU de Nancy, F-54000 Nancy, France

**Keywords:** velocity measurement, magnetic resonance imaging, articulatory motion, phase-contrast MRI

## Abstract

We evaluate velocity of the tongue tip with magnetic resonance imaging (MRI) using two independent approaches. The first one consists in acquisition with a real-time technique in the mid-sagittal plane. Tracking of the tongue tip manually and with a computer vision method allows its trajectory to be found and the velocity to be calculated as the derivative of the coordinate. We also propose to use another approach—phase contrast MRI—which enables velocities of the moving tissues to be measured directly. We recorded the sound simultaneously with the MR acquisition which enabled us to make conclusions regarding the relation between the movements and the sound. We acquired the data from two French-speaking subjects articulating /tata/. The results of both methods are in qualitative agreement and are consistent with other reviewer techniques used for evaluation of the tongue tip velocity.

## 1. Introduction

Since kinematics of the tongue defines most of the sounds in human speech, study of its motion is particularly interesting. Studying tongue motion and velocity patterns can be especially significant for better understanding the production of consonants in normal speech, and pathological speech as well, e.g., for estimation of the potential success of glossectomy [1].

There are multiple measurement methods of the positions and velocities of the upper articulators. In early years, techniques based on X-rays, such as cineflurography, represented an invaluable tool for speech research [2]. Another method, X-rays microbeam imaging, allows the kinematics of the upper vocal tract to be investigated [3] and velocities inside it to be evaluated [4]. However, due to influence of ionizing radiation, the allowed duration of the experiment is short, which significantly restricts the area of possible investigations. Ultrasound imaging provides insights on the tongue shape during speech [5,6] and thus can be used for velocity measurements [7]. However, ultrasound fails to visualize articulators separated from the probe by air, for example the tongue tip, and thus narrows horizon of the studies. Electromagnetic articulography allows the tracking of sensors glued on the upper vocal tract organs, and is widely spread today in speech sciences [8,9]. This technique uses several transmitters to create alternating magnetic fields at different frequencies which create electrical current in small coils used as sensors, attached to the articulators [10]. Combination of different frequencies with different magnitudes in the magnetic fields allows the recovery of the sensor position along time. It is also possible to evaluate velocities of the articulators using this method [11,12]. A device measuring distances from the tongue to the hard palate [13] was developed specifically for measurements of the tongue position and velocity.

Nowadays, magnetic resonance imaging (MRI) holds one of the leading positions in articulatory phonetics: it provides a fully non-invasive way of acquisition and has no known health risks. However, imaging of a speaking subject is a challenging problem since motion of certain articulators can be so fast that there is no possibility to achieve reasonable spatio-temporal resolution with conventional MRI techniques. This issue can be overcome with cine-MRI which was initially proposed for cardiac studies. Application of cine-MRI to speech investigations was proposed in [14] and commonly employed by a number of applications in speech studies in 2D [15,16,17] and in 3D [18,19]. In this cine method, periodical triggering pulses followed by sound tones are used. The subject is instructed to repeat target words after the tone, and the reconstruction is performed by gathering data corresponding to the same phase of the repetitive speech from different cycles into one image, which leads to sufficient improvement of spatio-temporal resolution. Application of cine-MRI requires several precise repetitions; however, natural non-periodicity of the speech leads to incorrect encoding of the information, and, as a consequence, to image artifacts [14,18]. Thus, the data should be carefully selected.

Usage of non-Cartesian sampling schemes can improve quality of the dynamic imaging and allow good spatio-temporal resolution without requiring several repetitions. It was shown that real-time MRI spiral sequences result in acceptable quality of the image series and can be used for speech studies [20]. Reconstruction of highly undersampled data acquired with radial schemes by regularized nonlinear inversion [21] allows the Nyquist limit to be overcome. It has been already used in a number of applications, including research on speech production [22] and tongue motion for horn players [23].

In order to calculate velocity of the tongue tip from cine-MRI or real-time sequences, it is necessary to track its position. This problem can be resolved with the HARP (harmonic phase) technique [24], which was initially developed for heart motion registration and was successfully applied to speech research [25,26,27]. In this approach, SPAMM (spatial modulation of magnetization) [28] or CSPAMM (complementary spatial modulation of magnetization) [29] is used to create tagged images. However, despite its interest, this technique has some weak points [30]. In particular, sometimes tracking errors happen due to limited band-pass filtering and damping of the tag pattern with the $T_{1}$ time constant [31].

The development of computer vision algorithms opens the possibility of tracking the articulators automatically without tagging, using various methods, including optical flow [20,32], shape and appearance models [33,34], and deep learning methods, such as convolutional neural networks [34], and, more recently, conditional generative adversarial network for fully automatic segmentation [35]. Commonly, to estimate velocity, all these methods estimate a displacement by tracking tissues from one image to the next, and then calculate a time derivative. A mid-sagittal plane is used (with inter-frame time differences of the order of 20–50 ms), and, in most cases, require some labeling supervised by a human.

While studying moving tissues, it is important to understand that conventional MRI techniques suppose that tissues do not move. However, if there is a motion, like in case of speech articulation, one can observe artifacts on images which can manifest in the form of blurring or signal loss. This is explained by the fact that spins position is encoded with their accumulated phase and their frequency of precession. In case a spin moves while the gradient fields are on, its accumulated phase does not correspond anymore to its position. This leads to changes in the magnitude and phase of the resulting data. Nevertheless, this effect which seems to impact negatively on the quality of images turns out to be useful for extraction of the motion information. It was shown [36] that usage of so-called bipolar gradient field (two consecutive magnetic field gradients of opposite polarity) in the direction of the motion allows acquisition of a phase proportional to the velocity of moving spins. Bipolar gradients are often characterized by the aliasing velocity parameter (VENC, or velocity encoding) [37], which should be chosen so that velocities of moving spins do not overcome its value in both directions. A phase of the reconstructed data can be used for the velocity determination, so that −π corresponds to—VENC and π corresponds to VENC. Created images have phase contrast.

Despite multiple studies discussed above and devoted to the velocity measurement, MRI is not widely used for evaluation of the velocity of the articulatory organs. Moreover, to our knowledge, there has been no attempts to use phase-contrast MRI for these purposes. In this paper, we propose to use two complementary approaches of MRI for evaluating the tongue tip velocity: velocity derivation from trajectory based on tongue tip registration in the mid-sagittal plane, and, directly, using phase contrast MRI sequences. For the latter one, accurate choice of the slice during the MRI acquisition allows the velocity curve to be found for some types of atriculatory motion without tracking, keeping the same region of interest during all the time series, while the former one has higher temporal resolution. With these two methods, this paper assesses consistency of the tongue tip velocity measurements with MRI modality. For alignment of images with the corresponding sound, speech of the subjects was recorded at the same time with the MRI acquisition.

## 2. Materials and Methods

Subjects include one male (27 years old) and one female (25 years old). Both subjects are native speakers of French with no speech or hearing problems. Subjects were asked to repeat /tata/ (with the last syllable stressed) periodically with the period of 2 s. The choice of the target utterance is also motivated by the fact that variability of the articulation for the sound /t/ is very small [38]. In case of this syllable, the tongue contacts the hard palate in the alveolar ridge region and then goes down, so that its trajectory corresponds to back and forth motion.

The data were acquired on a Siemens Prisma 3T scanner (Siemens, Erlangen, Germany) installed in Nancy Central Regional University Hospital under the approved ethics protocol “METHODO” (ClinicalTrials.gov Identifier: NCT02887053).

The protocol of acquisition comprised three sequences: a localizer, a real-time MRI sequence [21] with a slice placed in mid-sagittal orientation (TR = 2.22 ms, TE = 1.47 ms, number of radial spokes = 9, slice thickness = 8 mm, flip angle = 5 degrees, FOV = 220 mm × 220 mm, resolution of the resulting images is 136 × 136) and a phase-contrast sequence (Siemens BEAT_FQ). For the real-time sequence, we had four repetitions of the target utterance that resulted in a dynamic series of 400 images. Siemens BEAT_FQ is a Siemens implementation of a phase contrast cine-sequence [39] created for flow quantification in angiography. It results in a dynamic series of velocity-encoded phase contrast and magnitude 2D images. The sequence was triggered with the generator of a Signal Analyzer and Event Control system (SAEC) which was designed previously [40], and uses a period of 2 s. To achieve periodicity of the speech, the subject was asked to follow the stimuli (sound beep signals and red flashing screen). Parameters of the sequence were: temporal resolution = 35.52 ms (TR is 5.92 ms, number of lines per segment is 3, and two types of the velocity encoding gradient are used for the phase-contrast sequences so that 35.52=5.92×2×3), TE = 3.73, flip angle = 20 degrees, FOV = 220 mm × 220 mm, slice thickness = 6 mm, iPAT = 2, resolution of the resulting images is 196 × 196. The threshold velocity was chosen as VENC = 30 cm/s, according to the values of the tongue velocity from the literature [11]. The velocity encoding direction was chosen to be perpendicular to the slice, with the positive values corresponding to a movement in direction from feet to head. The total duration of the sequence was slightly more than 42 s, and the acquisition of a dynamic series of 44 images required 21 repetitions. Duration of one dynamic series was 44×35.52=1.56 s and its beginning corresponds to the trigger signal. For determination of time for each image obtained with the cine-MRI sequence, we used trigger time from the DICOM header.

Following the goal to minimize tangential motion of the tongue tip, position of the slice for the phase-contrast sequence should be perpendicular to this trajectory. Positioning of the correct plane can be done using an additional real-time sequence. Moreover, the images acquired in the mid-sagittal plane can serve as an independent method of the tongue tip velocity determination. Therefore, the chosen protocol contained a localizer, a real-time MRI sequence in the mid-sagittal plane and a phase contrast sequence (PC cine-MRI) in an oblique plane defined from the previous real-time sequence. We used a cine-sequence to improve the spatio-temporal resolution.

**Figure 1:**
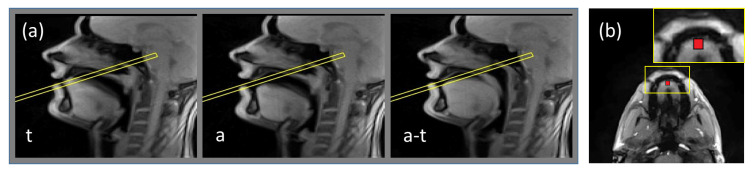
Illustration of the routine of the slice and region of interest positioning. (**a**) examples of frames of dynamic acquisition of the subject obtained with the real-time MRI sequence in the mid-sagittal plane: during production of /a/, of /t/ and transition between /a/ and /t/. The frame denotes the position of the slice touching the hard palate and perpendicular to the tongue motion direction; (**b**) example frame of the acquisition in the plane denoted by the frame in Figure 1a. Region of interest is shown by the rectangle. The inset presents zoomed-in region containing ROI.

In order to define the slice position for the phase-contast sequence, all images of the series acquired in the mid-sagittal plane of the subject articulated /tata/ were displayed on the scanner console, and the slice for the phase contrast sequence was chosen to be touching the hard palate and perpendicular to the tongue motion direction. During the acquisition with the phase contrast sequences, the subject was articulating the same utterance /tata/ at the same rate and articulation. The resulting dynamic series was used for determination of the region of interest (ROI) corresponding to the tongue tip, which was chosen to be a 5 × 5 pixel square (5.6 × 5.6 mm^2^. Examples of the slice position and of the region of interest are shown in Figure 1.

Audio was recorded simultaneously with imaging at a sampling frequency of 16 kHz by using an optoacoustics fibre-optic microphone (FOMRI III, Optoacoustics Ltd., Mazor, Israel) placed in the scanner. To remove acoustic noise of the MRI machine, we used the denoising algorithm proposed in [41]. In order to obtain clear samples of the speech, the subject was asked to repeat the target utterance 2–3 times before and after the imaging sequence was played.

Further sound processing, such as calculation of the sound level and the spectrogram, was done in MATLAB using standard functions. In order to compare velocity curves with the sound, it was necessary to define specific points. The natural choice was: onset of the first vowel ($q_{1}$), offset of the first vowel ($q_{2}$), onset of the second vowel ($q_{3}$) and offset of the second vowel ($q_{4}$) since they are clearly visible from both the acoustic signal and the spectrogram. These points were found semi-automatically using the following simple algorithm. The denoised sound was filtered with a filter finding the maximal value, and then with a median filter (both of 10 ms). Then, if a segment contains only values greater than background level and is longer than 0.1 s, it was treated as speech or beep signal. In our work, the level of speech is always greater than that of beep signals, so we compared mean values of the segments for separating these two types of signals. Threshold values depend on subjects and sequences. Resulting values were corrected manually using the spectrogram, using the fact that vowels correspond to a strong signal between 0 and 4000 Hz, and can thus be easily separated from the consonants or the noise.

In order to align the sound with MRI data, we used SAEC. We applied it to record timestamps of the sequence endings and of the beep and flash generator signal. The latter was also recorded into the TTL channel of the opto-acoustic system. The first step of the alignment was the detection of the sequence noise endings in audio recordings that can be easily done by thresholding. Then, knowing the duration of the MRI sequence, it was easy to calculate the starting point. Final adjustment was done also automatically using comparison of timestamps of the generator with those recorded into the TTL channel.

For evaluation of the tongue tip velocity from images in the mid-sagittal plane, a tool was developed in MATLAB. For the pre-processing of an image I(x,y) before the tracking, we used the filtering algorithm proposed in [42] with the following parameters: we assumed the background was 10% of the maximal intensity $I_{max}=\max\left(I\left(x,y\right)\right)$ for the operation of background subtraction: $I^{\prime }\left(x,y\right)=I\left(x,y\right)-0.1I_{max}$, the thresholding parameter was 10% of the maximal intensity after the background subtraction $I_{max}^{\prime }=\max\left(I^{\prime }\left(x,y\right)\right)$: $I^{\prime \prime }\left(x,y\right)=\left\{I^{\prime }\left(x,y\right),\;if\;I^{\prime }\left(x,y\right)>0.1I_{max}^{\prime };\;0,\;if\;I^{\prime }\left(x,y\right)<0.1I_{max}^{\prime }\right\}$, size of the median filter was 3 × 3, edge strength was 0.1, minimal edge distance was 3 pixels, size of the Gaussizan filter was 5 × 5, and its radius was 5. After the filtering, most of the motion artifacts were removed, as well as the textures information, which simplifies further motion registration.

**Figure 2:**
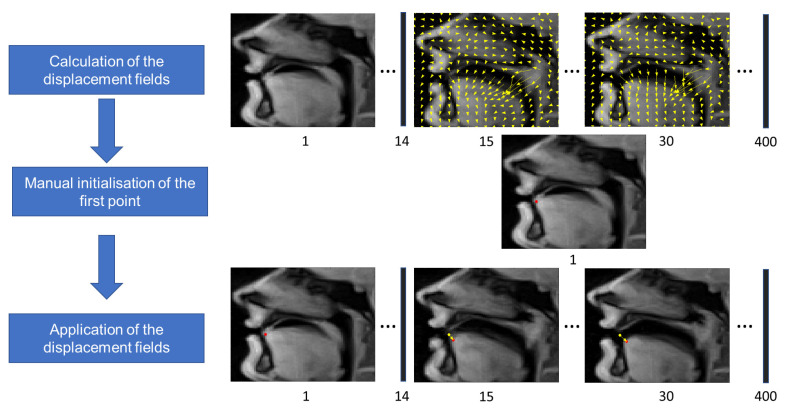
Scheme of the tongue tip tracking procedure illustrated with fragments of images for S1 acquired in the mid-sagittal plane. The yellow arrows show the displacement fields and the numbers under the images denote frame numbers. The red circles are the resulting points, and the yellow circles show the manually selected first point given for the comparison.

For motion registration, we used the method proposed in [43]. It consists in minimizing a cost function C(u)=S(u)+λR(u), where *u* is a displacement field, S(u) is a similarity function which was chosen to be the sum of squared pixel intensity differences, and $R\left(u_{x},u_{y}\right)=∥\left(G_{x},G_{y},G_{t}/v_{0}\right)u_{x}{∥}^{2}+{∥\left(G_{x},G_{y},G_{t}/v_{0}\right)u_{y}∥}^{2}$ is a regularization term imposing spatial and temporal smoothness on displacement field, where $G_{x},G_{y}$, and $G_{t}$ are sparse matrices representing the gradients in the space and time directions and $v_{0}$ is a parameter allowing tuning of the relative weight given to smoothness in space and smoothness in time. The displacement fields are presented with vectors with length $N=N_{x}N_{y}N_{t}$, and the gradients thus represent sparse matrices of size N×N. The resulting nonlinear least-squares optimization problem was solved using a multiresolution Gauss–Newton scheme. Tongue tip was selected manually by the user in the first frame, and propagated to the next frames using the displacement fields. The illustration of the tongue tip tracking procedure is shown in Figure 2.

To enable the comparison with another method, the tongue tip position was tracked fully manually for the whole non-denoised temporal set of the data acquired in the mid-sagittal plane. Since during the fast phases of the tongue motion real-time MRI images exhibit motion blurring, there is some ambiguity in finding the exact position of the tongue tip. In order to minimize subjectivity of the labeling, its position was selected three times, and then resulting values were averaged. The comparison with the manually labeled tongue positions allowed us to determine that optimal regularization parameter is λ=0.01.

To be able to compare resulting values with those obtained with phase-contrast MRI, velocities were projected on the direction perpendicular to the plane. For that, information about position and orientation of the patient from DICOM files was used to define the relative position of the slices. The derivative of the distance between the tongue tip point and the line denoting the upper edge of the slice was defined as the transverse velocity. We define positive direction of the displacement to be up, and, consequently, positive direction of the velocity to be upwards.

Evaluation of velocity from images obtained with the phase-contrast sequence was done by averaging values inside the ROI without application of additional filters. To remove influence of the low density regions, we did not take into account phase of the pixels having intensity lower that 5% of the maximal image intensity. Conversion from 16-bit DICOM images to true phase-contrast images was done by taking into account the fact that Siemens uses 12 bit for the information encoding, thus one can use the formula $I_{\left(-\pi ,\pi \right)}=-\pi +I_{16bit}/\left(2^{12}-1\right)*2\pi$. Conversion from phase to speed can be done using the formula $I_{\left(-VENC,VENC\right)}=I_{\left(-\pi ,\pi \right)}\cdot VENC/\pi$.

To find points where velocity takes zero value, we used linear interpolation of the data. We denote these points as follows: the starting point of the movement $t_{0}$, first extreme top point $t_{1}$, first extreme low point $t_{2}$, second extreme top point $t_{3}$, second extreme low point $t_{4}$, and stopping point of the movement $t_{5}$. Velocity peaks were defined as the extreme values between the selected points (in between of $t_{1}$ and $t_{2}$, $t_{2}$ and $t_{3}$, $t_{3}$ and $t_{4}$ for each cycle independently), and mean values of different phases of the utterance were calculated as the mean value in between the same points.

## 3. Results

**Figure 3:**
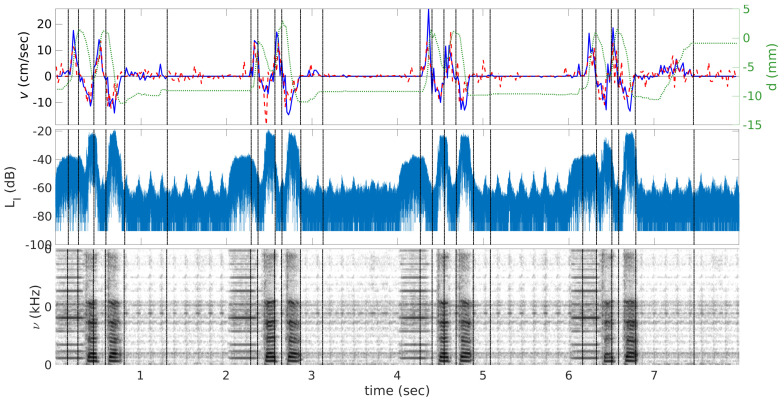
Velocity *v* (time) (the left scale) and displacement curves *d* (time) (the right scale) are shown on the top, sound level $L_{I}$ (time) (middle) and sound spectrogram *s* (time, ν) (bottom) extracted from the real-time MRI images of the subject S2 and corresponding sound recordings. The solid curve corresponds to velocities evaluated manually, the dashed curve corresponds to those evaluated automatically, and the dotted curve corresponds to the displacement between the lower boundary of the slice used for the phase-contrast sequences and the tongue tip. Positive velocity values correspond to closure of the mouth and negative to opening. Vertical dash-pointed lines denote time when velocity takes zero value.

An example velocity curve extracted from real-time acquisitions in the mid-sagittal plane is shown in Figure 3 (see also Supplementary Materials, Videos S3 and S4). One can see that, in general, automatic labeling is in agreement with the manual one; however, application of the automatic method leads to a higher variability of the peak values. These differences are also reflected in Table 1.

Corresponding sound levels and sound spectrograms are plotted in the same figure. Beep signals used for synchronization of subject’s utterances are also visible on the sound recordings. Both subjects repeated /tata/ with a slight delay after the beep, so that the first, less intensive peak on the acoustic signal corresponds to the beep, and the following two correspond to vowels /a/.

**Figure 4:**
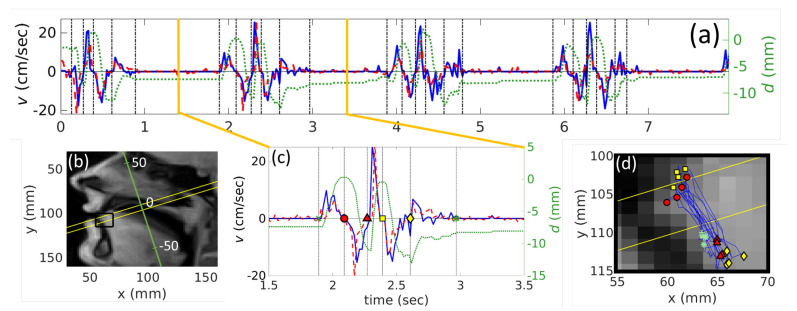
Results for S1 articulating /tata/ calculated from real-time MRI. (**a**) the whole velocity *v* (time) (the left scale) and the displacement *d* (time) curves (the right scale). The solid curve denotes velocity found manually, the dashed curve corresponds to automatically evaluated velocities, and the dotted line corresponds to the tongue tip displacement. The solid bold vertical lines present the borders of the Figure 4c. Positive velocity values correspond to the upward tongue tip movement to achieve the constriction; (**b**) part of an image taken in the mid-sagittal plane, with the region of interest, shown in Figure 4c (solid black rectangle) and the displacement axis (the inclined axis); (**c**) example of one repetition of /tata/. Vertical dash-dotted black lines indicate points $t_{0}-t_{5}$ where velocity becomes zero and are denoted as the *x* ($t_{0}$), the circle ($t_{1}$), the triangle ($t_{2}$), the square ($t_{3}$), the diamond ($t_{4}$) and the asteriks ($t_{5}$); (**d**) the whole trajectory of the tongue motion on an image in the mid-sagittal plane with the same markers as for Figure 4c corresponding to $t_{0}-t_{5}$.

Points where velocity takes zero value are of interest because these points correspond to extreme points on the trajectory of the tongue motion. To verify that, we plot these stop-points on the trajectory map (see Figure 4). Indeed, for both subjects, points of zero velocity correspond to the extreme points of the tongue tip trajectory. In addition, we verified that the absolute values of full velocities are sufficiently close to the absolute values of the projections calculated as described in Materials and Methods. The difference was less than 2% everywhere on the curve during speech (from $t_{1}$ to $t_{4}$), which means that the tongue tip trajectory was almost perpendicular to the selected plane, which is also confirmed by trajectory plots (see Figure 4).

From Figure 3, it can be seen that stop-points $t_{1}-t_{4}$ are before the points $q_{1}-q_{4}$ from the sound recordings. Means and standard deviations of difference between these two sets of points are presented in Table 2. This means that the points where velocity cancels out, i.e., points of articulatory stability, are approximately in the middle of the sounds /t/ and /a/ as expected.

**Figure 5:**
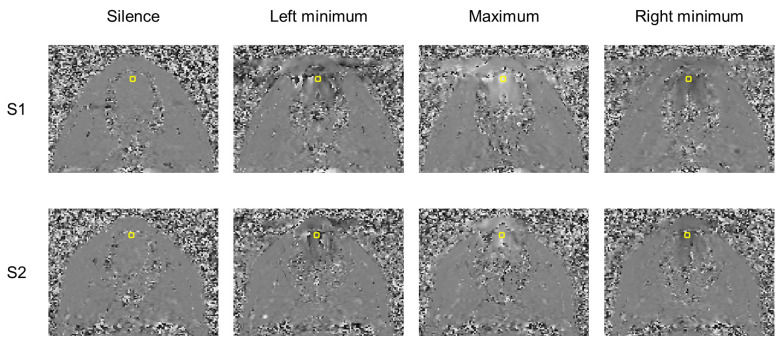
Examples of phase-contrast images acquired in the oblique plane for the initial position and the extreme tongue trajectory points which are presented in Table 1 for both subjects. Black color corresponds to −π and motion downwards and white corresponds to π and upwards. The yellow rectangle denotes the region of interest.

Examples of the acquisition with phase-contrast cine-MRI sequence are shown in Figure 5. Corresponding videos are presented in Supplementary Materials (see Videos S3 and S4). It can be seen that some artifacts in phase-encoding direction (left-right) take place due to the non-periodicity of the motion. However, they affect mostly lips.

**Figure 6:**
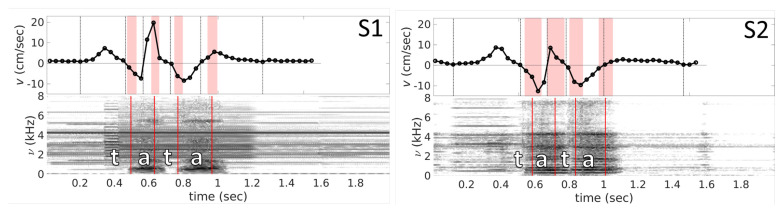
Velocity curves *v* (Time) and spectrograms *s* (Time, ν) obtained from PC cine-MRI for subjects S1 and S2. Vertical dashed black lines on the velocity plots indicate time when velocity takes zero value, and denoted in correspondence with Figure 4. The filled zones indicate $\left(\mu_{i}-\sigma_{i},\mu_{i}+\sigma_{i}\right)$ for onset and offset of the both vowels, where μ is corresponding mean, σ is the corresponding standard deviation, and *i* is index of the zone. The spectrograms are obtained from the sound averaged on the 21 repetitions aligned with the MR images. The vertical solid lines denote averaged onsets and offsets for both vowels.

**Figure 7:**
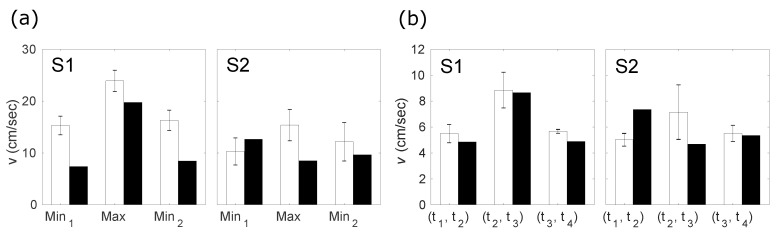
(**a**) peak velocity values evaluated with real-time MRI with manual labeling (white bars) and PC cine-MRI (black bars). Height of the bars represents mean absolute values of the first minimum, the maximum between two minima, and the second minimum. Error bars correspond to the standard deviation; (**b**) mean velocity values evaluated with real-time MRI with manual labeling (white bars) and PC cine-MRI (black bars). Height of the bars represents mean absolute values of the first opening having place within interval $\left(t_{1},t_{2}\right)$, the closure between two openings (within $\left(t_{2},t_{3}\right)$), and the second closure (within $\left(t_{3},t_{4}\right)$). Error bars correspond to the standard deviation.

Plots obtained from PC cine-MRI (Figure 6) demonstrate, in general, good agreement with those obtained from indirect velocity calculations. One can see that both types of sequences produce velocity curves that have pronounced negative peaks and a positive peak between them. Similar to the graphs extracted from mid-sagittal plane images, feature points for the velocity curves $t_{1}-t_{4}$ are slightly before the sound feature points $q_{1}-q_{4}$ which can be seen from Table 3 and Figure 6. In addition, one should note the existence of velocity plateaus in the vicinity of the consonants /t/, as it expected. This is explained by the fact that at this moment the tongue tip touches the hard palate in the alveolar region and rests there during the stop closure. However, there is some quantitative difference in values of the velocity peaks (see Table 1) and mean velocities (see Table 4). Graphical comparison of the peak and mean velocities obtained with these two methods is presented in Figure 7.

In general, subject S2 demonstrates more variability in the articulation of /tata/, which is reflected in the values of the standard deviations for both the variability in time and in peak values (see Table 1, Table 2 and Table 3 and Figure 7). This variability can also be seen directly from Figure 6. In addition, one can see a clear positive peak for subject S2 before the vowel articulation (see Figure 3); S1 also did not demonstrate such a strong peak (example is in Figure 4). This is because the rest position of S2 between the utterances was more open than that of S1. S2 therefore needed a more pronounced tongue tip movement to reach the hard palate. This interpretation is supported by the displacement curves (see Figure 3 and Figure 4).

## 4. Discussion

This study compared two independent methods of the tongue tip velocity measurements in the context of speech production.

Real-time acquisition in the mid-sagittal plane does not depend on the ability of the subject to repeat the same utterance with a high precision and has a good time resolution (20 ms). In addition, the selection of the mid sagittal plane is easy enough and does not require any preliminary acquisition. However, fast motion produces artifacts that complicate the correct detection of the tongue tip. Moreover, tracking the tongue tip is a separate problem which requires either time for manual delineation, or the implementation of advanced algorithms. Even if the algorithm applied in this paper showed qualitative agreement with manual delineation, there is still a quantitative difference which cannot be removed with the regularization factor tuning.

Since MRI acquisition in a mid-sagittal plane does not provide a direct velocity measure and involves calculating the difference of the tongue position between two adjacent frames, it can be supplemented with phase contrast sequences. They enable the velocity to be evaluated directly, and without tracking. We verified that it is possible to choose an acquisition plane so that the tangential component of the velocity during the articulation is negligible. Thus, within this period, the tongue tip is always inside the selected ROI, and calculation of the velocity does not require any tracking. However, this method presents weak points. The plane should be chosen carefully: it is necessary to do a prior acquisition in the mid-sagittal plane and to set the plane inclination, transverse to the tongue motion, with great accuracy. In addition, probably partial volume effects have some impact on the velocity value because, at certain moments, especially close to the lowest point of the tongue tip trajectory, the acquisition slice is mostly filled by the air, but not by the tongue tissue. Despite the air possibly having a velocity different from the tongue tip, it produces signal with very low magnitude and thus this influence on the phase should be small.

For the proposed method, it is especially important to pay attention to the time resolution. The phase contrast cine-MRI enables obtaining one speech cycle that contains information from multiple repetitions, which dramatically improves temporal resolution. However, application of cine-MRI requires a strict periodicity of the movements. One should understand that a signal acquired with MRI represents not lines of images, but lines of the data, which is, in the simplest case, a Fourier transform of the image data. Therefore, if the movements are not periodical, distortions in the signal space do not lead to easily predictable distortions in the image space. By analyzing the sound recordings, we found that both subjects failed to articulate the utterance /tata/ with a high periodicity. As a result, velocity curves exhibit values which differ from those obtained from the images acquired in mid-sagittal plane. Moreover, the analysis of rates during different speech phases has shown that the difference is more significant for subject S2 which shows much higher variability of the key time points position extracted from the sound recordings. Nevertheless, there is a qualitative agreement between the two methods.

One of the challenges of this paper also was to verify that possible out-of-plane motion does not affect the results. From the Videos S3 and S4 (see Supplementary Materials), it can be seen that the phase is always well-defined inside the ROI which means that there are always tongue tissues. Despite it being seen from Figure 4 that sometimes the point, chosen to denote the tongue tip in the mid-sagittal plane, can move outside the oblique acquisition plane used for the PC cine-sequence, we can suppose that, if the displacement is small, velocity of the surrounding tissues does not differ from the target point much. To verify this hypothesis, one can compare positions of the points $t_{2}$ and $t_{4}$ that correspond to the opened mouth position during the phonation of /a/. Comparison of Table 2 and Table 3 shows that these stop-points indeed correspond to each other within standard deviation for both subjects; thus, the hypothesis can be confirmed.

Further improvement of the acquisition protocol such as usage of other stimulation signals, a longer cycle period, or other target utterances may help subjects to articulate with a higher periodicity, which would lead to the reduction of artifacts.

As a future prospective, another possibility to measure velocities within the proposed method is to use phase contrast real-time sequences [44,45]. With this approach, the subject does not need to be strictly periodic, which substantially simplifies the task. However, it will require accurate adjustment of the sequence parameters to reach a sufficient quality.

Finally, there is a possibility to use in-plane velocity encoding, so that the tongue tip is always in plane, and the velocity is measured with a direct method. However, it will still require tongue tip tracking.

The recovered tongue velocities are on the same order of magnitude as those given by the literature. For example, in [11], subjects produced the utterance “a bad daba” and the velocity was measured with electromagnetic articulography for a point 1 cm posterior to the tongue tip. Measures ranged from 4 to 12 cm/s, while mean velocities in our research took values of 5–9 cm/s. Similarly, our results are in good agreement with those obtained with the method proposed in [13] and with curves obtained with the X-ray microbeam technique [4].

## 5. Conclusions

This study was devoted to the evaluation of the tongue tip velocity with two different MRI techniques: real-time MRI and phase-contrast cine-MRI. Real-time acquisition in a mid-sagittal plane enables velocity of the tongue tip to be calculated from its position on two adjacent frames. From comparison with the sound recorded simultaneously with the MRI, we have found that these stop-points are before the onset and offset of both vowels, as expected.

The present investigation is the first to use phase contrast sequences for measuring articulators’ velocity. Our results for phase-contrast cine-MRI from two subjects have shown a qualitative agreement with conventional acquisition in the mid-sagittal plane. It was shown that, due to the sensitivity of this technique to non-periodicity of the target utterance, values of the velocity are somewhat distorted. Further work in this direction may improve reliability of this promising approach so as to enable kinematics of the tongue and other articulatory organs to be investigated with safe and non-invasive MRI technique without complex post-processing.

## Figures and Tables

**Table 1:** Mean (std. dev.) of the peak velocity values.

Subject	Source	Left Minimum (cm/s)	Maximum (cm/s)	Right Minimum (cm/s)
	Manual	−15.29 (1.80)	23.89 (2.06)	−16.30 (1.96)
S1	Automatic	−18.25 (3.69)	16.89 (7.51)	−11.48 (2.84)
	PC cine-MRI	−7.38	19.74	−8.43
	Manual	−10.28 (2.61)	15.37 (3.02)	−12.16 (3.70)
S2	Automatic	−12.09 (4.48)	13.99 (2.02)	−10.78 (1.45)
	PC cine-MRI	−12.66	8.48	−9.66

**Table 2:** Mean (std. dev.) of time difference between feature points extracted from real-time MRI data in the mid-sagittal plane ($t_{1}-t_{4}$) and from the corresponding sound ($q_{1}-q_{4}$). Time resolution of the MRI is 19.8 ms.

	$q_{1}-t_{1}$ (ms)	$q_{2}-t_{2}$ (ms)	$q_{3}-t_{3}$ (ms)	$q_{4}-t_{4}$ (ms)
S1	65 (10)	64 (5)	60 (22)	68 (17)
S2	11 (49)	12 (43)	12 (21)	11 (13)

**Table 3:** Mean (std. dev.) of time difference between feature points extracted from PC cine-MRI data ($t_{1}-t_{4}$) and from the corresponding sound ($q_{1}-q_{4}$). Time resolution of the MRI is 35.52 ms.

	$q_{1}-t_{1}$ (ms)	$q_{2}-t_{2}$ (ms)	$q_{3}-t_{3}$ (ms)	$q_{4}-t_{4}$ (ms)
S1	37 (27)	71 (22)	48 (24)	68 (28)
S2	73 (49)	50 (50)	59 (39)	12 (41)

**Table 4:** Mean values of the velocity of the tongue tip during different phases of the speech.

Subject	Source	First, Opening	Closure	Second Opening
		(cm/s)	(cm/s)	(cm/s)
	Manual	5.50	8.85	5.67
S1	Automatic	4.65	5.41	4.06
	Cine-MRI	4.85	8.65	4.89
	Manual	5.02	7.15	5.51
S2	Automatic	4.20	5.13	4.55
	Cine-MRI	7.36	4.68	5.35

## Supplementary Materials

The following are available at https://www.mdpi.com/2313-433X/6/5/31/s1, Video S1: Video with sound of speaking S1 acquired in the mid-sagittal plane with the real-time sequence. The vertical red line at the plots on the right corresponds to the current position. Video S2: Video with sound of speaking S2 acquired in the mid-sagittal plane with the real-time sequence. The vertical red line at the plots on the right corresponds to the current position. Video S3: Video of speaking S1 acquired in the oblique plane with the phase-contrast cine-sequence. The white rectangle corresponds to the region of interest. Video S4: Video of speaking S2 acquired in the oblique plane with the phase-contrast cine-sequence. The white rectangle corresponds to the region of interest.

## Abbreviations

The following abbreviations are used in this manuscript:

MRI	Magnetic resonance imaging
PC	Phase contrast
SAEC	Signal analyzer and event control system
VENC	Velocity encoding
